# Study of Melatonin as Preventive Agent of Gastrointestinal Damage Induced by Sodium Diclofenac

**DOI:** 10.3390/cells9010180

**Published:** 2020-01-10

**Authors:** Aroha B. Sánchez, Beatriz Clares, María J. Rodríguez-Lagunas, María J. Fábrega, Ana C. Calpena

**Affiliations:** 1 Department of Pharmacy and Pharmaceutical Technology, Faculty of Pharmacy and Food Sciences, University of Barcelona, 08028 Barcelona, Spain; aroha_89_1@hotmail.com (A.B.S.); anacalpena@ub.edu (A.C.C.); 2 Department of Pharmacy and Pharmaceutical Technology, Faculty of Pharmacy, University of Granada, 18071 Granada, Spain; 3 Department of Biochemistry and Physiology, Faculty of Pharmacy and Food Sciences, University of Barcelona, 08028 Barcelona, Spain; mjrodriguez@ub.edu (M.J.R.-L.); mjfabrega.f@gmail.com (M.J.F.)

**Keywords:** melatonin, NSAIDs, gastric injuries, antioxidant

## Abstract

Safety profile of nonsteroidal anti-inflammatory drugs (NSAIDs) has been widely studied and both therapeutic and side effects at the gastric and cardiovascular level have been generally associated with the inhibitory effect of isoform 1 (COX-1) and 2 (COX-2) cyclooxygenase enzymes. Now there are evidences of the involvement of multiple cellular pathways in the NSAIDs-mediated-gastrointestinal (GI) damage related to enterocyte redox state. In a previous review we summarized the key role of melatonin (MLT), as an antioxidant, in the inhibition of inflammation pathways mediated by oxidative stress in several diseases, which makes us wonder if MLT could minimize GI NSAIDs side effects. So, the aim of this work is to study the effect of MLT as preventive agent of GI injury caused by NSAIDs. With this objective sodium diclofenac (SD) was administered alone and together with MLT in two experimental models, ex vivo studies in pig intestine, using Franz cells, and in vivo studies in mice where stomach and intestine were studied. The histological evaluation of pig intestine samples showed that SD induced the villi alteration, which was prevented by MLT. In vivo experiments showed that SD altered the mice stomach mucosa and induced tissue damage that was prevented by MLT. The evaluation by quantitative reverse transcription PCR (RT-qPCR) of two biochemical markers, COX-2 and iNOS, showed an increase of both molecules in less injured tissues, suggesting that MLT promotes tissue healing by improving redox state and by increasing iNOS/NO that under non-oxidative condition is responsible for the maintenance of GI-epithelium integrity, increasing blood flow and promoting angiogenesis and that in presence of MLT, COX-2 may be responsible for wound healing in enterocyte. Therefore, we found that MLT may be a preventive agent of GI damages induced by NSAIDs.

## 1. Introduction

**Figure 1:**
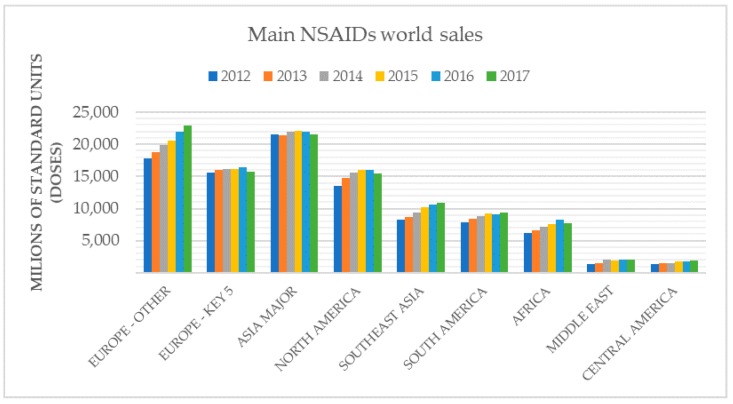
IQVIA^®^ (IQVIA Inc., Parsippany, NJ, United States) data about the main nonsteroidal anti-inflammatory drugs (NSAIDs) world sales from 2012 to 2017, including Europe (Key 5 are Germany, France, Italy, Spain, and Britain), Asia, North, South, and Central America, Africa, Middle East, and Southeast Asia.

Nonsteroidal anti-inflammatory drugs (NSAIDs) world sales of last year confirm that they still are one of the most consumed drugs (Figure 1). The safety profile of NSAIDs has been widely studied. Therapeutic and side effects at the gastric and cardiovascular level have been popularly associated with the inhibitory effect on endoperoxide-H synthase1 and -2 (PGHSs) also known as cyclooxygenase enzymes (COX).

**Figure 2:**
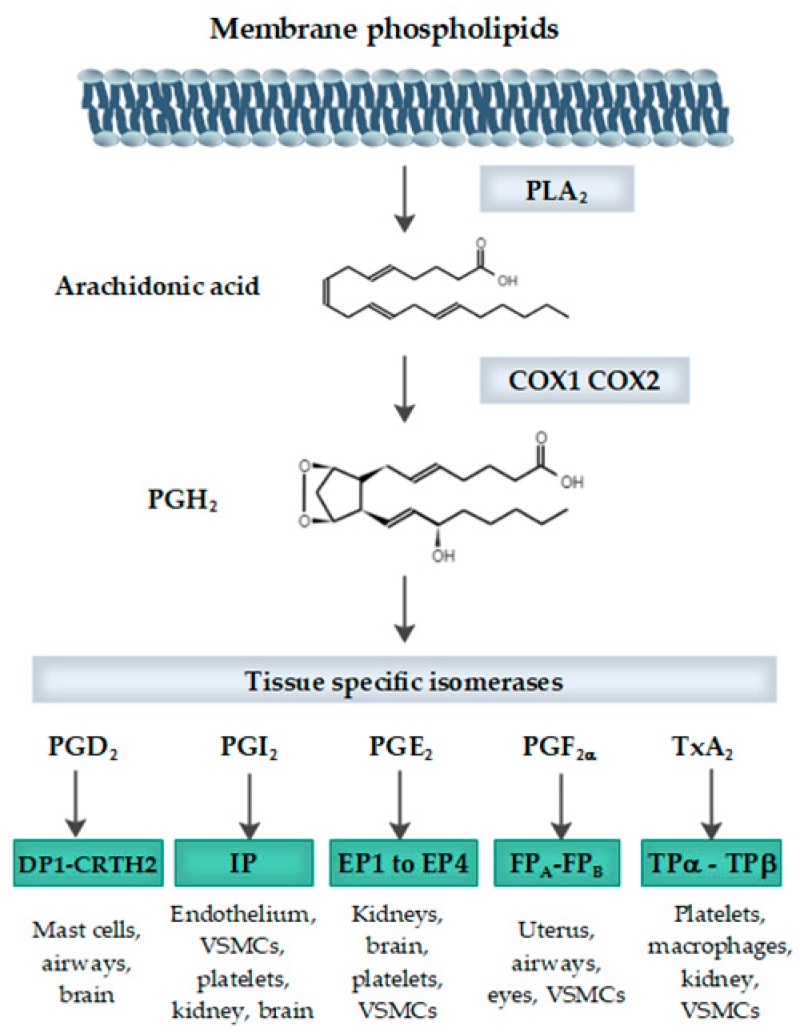
Synthesis of prostaglandins scheme from membrane phospholipids, including enzymes, substrates, and receptors. PLA_2_ (phospholipase A2); COX1 (cyclooxygenase 1); COX2 (cyclooxygenase 2); PGH_2_ (prostaglandin H2); PGD_2 (_prostaglandin D2); DP1 and CRTH2 (DP2) (D-prostanoids receptors); PGI_2_ (prostaciclin); IP (prostacyclin receptor); PGE_2_ (prostaglandin E2); EP1 to EP4 (prostaglandin receptors); PGF_2α_ (prostaglandin F2α); FP_A_ and FP_B_ (prostanoid receptors, isoform A and B); TxA_2_ (thromboxane A2); and TPα and TPβ (thromboxane prostanoid receptors). Figure edited with Edraw^TM^Max 9.4 (Edrawsoft, Hong Kong, China, 2019).

Two isoforms of COX have been characterized, the constitutive COX (COX-1), which is expressed in most mammalian tissues, and the inducible COX (COX-2) [1]. Both catalyze the oxidation of arachidonic acid (AA) to prostaglandin G2 (PGG_2_), which leads to prostaglandin H2 (PGH_2_) by the action of peroxidase, then the tissue-specific prostaglandin synthases lead to all the different prostaglandins, prostaglandin D2 (PGD_2_), prostaglandin F2α (PGF_2α_), prostaglandin E2 (PGE_2_), prostaciclin (PGI_2_), and thromboxane A2 (TXA_2_) [2] (Figure 2).

Classical outcomes associated COX-2 expression, with proinflammatory and pain events [3,4], Xie et al. were the first to demonstrate that *COX-2* gene expression is induced in inflammation conditions in 1991 [5], while COX-1 was described as protector of gastric mucosa since several authors had described that prostaglandins PGE_2_ had cytoprotective [6], antiulcer effects [7,8], and PGE_2_ synthesis was found to be more related to COX-1 than to COX-2 [9]. PGI_2_ was also described as a potent inhibitor of in vivo gastric acid secretion and enhancer of mucosal blood flow when infused intravenously [10].

It was widely accepted that the main cause of non-selective NSAIDs gastrointestinal (GI) damage, was the inhibition of COX-1 pathways, which triggered the research for selective inhibitors of COX-2 (COXIBS) [11]. Selective inhibitors of COX-2 seemed to have a safer GI profile than classical NSAIDs but over the time it was shown an increased risk of cardiovascular side effects, decreasing the benefit-risk balance of these drugs [12,13]. Among other reasons, the key-role of COX-2 in the synthesis of PGI2 is explained by the fact that it is an important vascular-protector with vasodilator, antithrombotic, antiaggregant, and anti-inflammatory properties [14]. In the mid-90s Langenbach et al. reported that inhibition of COX-1 does not cause spontaneous gastric damage [15,16], and some years later, new findings associated *COX-2* gene expression to wound healing in enterocytes via p38 mitogen-activated protein kinases (p38 MAPK) [17] evidencing that the concept of assigning homeostatic and pathological functions to COX-1 and COX-2 respectively was a plain approach.

Currently, there are evidences of the involvement of multiple cellular pathways in NSAIDs-mediated-GI damage. The specific sequence is unknown but could be initiated by the alteration of the protective gastric mucus layer [18] as result of the interaction of NSAIDs with phospholipids (PL) as phosphatidylcholine (PC) [19,20] the main component of gastric mucus layer and mucosa [21]. Studies proved that NSAIDs have a strong affinity to form ionic and hydrophobic, non-covalent and reversible associations with zwitterionic PL (specifically PC) [20]. The direct interaction between NSAIDs and PL together with the decreased mucus secretion by inhibition of PGE_2_, also mediated by the anti-inflammatory drugs [22], may lead changes in the fluidity, permeability, and biomechanical properties of cellular membrane of the gastric epithelium. The invasion of the intestinal mucosa by the enterobacteria would be responsible for the immune response, including adherence and infiltrate of leucocyte (neutrophils), when lipopolysaccharide (LPS) and other endotoxins are recognized by Toll-like receptor 4 (TLR-4), which via myeloid differentiation primary response 88 (MyD88) protein [23] results in the activation of nuclear factor Κβ (*NFΚβ*) target genes that are then responsible for inducible form of nitric oxide synthetase (iNOs) and COX-2 expression [24,25]. Neutrophils trigger the emergence of superoxide radicals (O2^.−)^ that react with nitric oxide (NO) leading in peroxynitrites (ONOO^−^), high reactive molecules responsible for protein oxidation, lipid peroxidation and enzymes inactivation [26], main origin of apoptosis and necrosis of gastric epithelium.

A parallel mechanism, also responsible for production of reactive oxygen species (ROS) and leucocyte infiltration that feeds the cycle of ONOO^−^ exacerbating the injuries, is a consequence of the inhibition of Glucose-6-phosphate dehydrogenase (G6 PDH) by NSAIDs, this increases the entry of pyruvate into the mitochondria, which finally, owing also to the inhibition of acyl -CoA carboxylase (ACC) by NSAIDs, leads to the uncoupling of mitochondrial oxidative phosphorylation [27]. The uncoupling of oxidative phosphorylation, which finally results in depleted adenosine triphosphate (ATP) levels [28,29], depends on the Pka (the negative base −10 logarithm of the acid dissociation constant (Ka)) of the NSAID, the lower the pKa value, the minimum concentration is required to uncouple oxidative phosphorylation [30], it also explains why the stronger the acid the higher the injuries.

The role of iNOs/NO depends on the epithelium redox state, under non-oxidative condition NO is an endogenous molecule responsible for the maintenance of GI-epithelium integrity, increasing blood flow and promoting angiogenesis via vascular endothelial growth factor (VEGF) pathways [31,32,33], but in the presence of O_2_^.−^, as explained before NO contributes to ONOO^−^ production. NO has been proved as protective molecule against NSAIDs-induced gastric ulceration by a mechanism independent of gastric acid secretion [34] but it is known that it also plays a role as an inhibitor in the regulation of gastric acid secretion [35].

Different studies showed a direct relation between acid and GI injuries induced by NSAIDs, in fact the main prescribed drugs to prevent such injuries are the proton-pump inhibitors (PPI) [36,37], Fornai suggests that beneficial effects of PPIs on mucosal injury are likely to be independent from the COX-2/PGE_2_/VEGF pathway [38] so considering all the involved cellular pathways it can be deduced that the intact mucus layer protects epithelium from the action of acid pepsin, protons, [H+] and, in addition, when the barrier is damaged, acid aggravates gastric injuries. There are also evidences of an increased basal gastric acid concentration mediated by NSAIDs due to the basal gastric fluid volume reduction [39].

Regardless of selective COX-2 inhibitors (COXIBS) manifested less GI damage [40], it is known that COX-2 has an essential role in enterocyte wound healing, through a mechanism related with NFΚβ via and p38MAPK-dependent histone 3 phosphorylation, which is an important component of the intestinal wound-healing response [17], additionally PGE_2_ participates in the differentiation of human goblet intestinal epithelial cells during homeostatic conditions [41] and PGI_2_ in angiogenesis [10]. Bile salts seems also be related with intestinal damage cause by NSAIDs by forming super-toxic micelles [42,43], injuries that could be avoided by PC [44] evidencing the role of mucus barrier as protective factor of GI mucosa from lesions generated by NSAIDs. 

Although, GI safety profile depends on the dose and kind of NSAID and a benefit-risk assessment strategy to select an anti-inflammatory drug has been described [45], GI events related to chronic NSAID consumption must be considered.

The use of PPI is highly accepted, different studies concluded that the efficacy and safety levels are acceptable [46] but a new trend, focused on the relationship between intestinal microbiota and several diseases, shows up that a pH change in the GI tract, would be related to a change in the microbiota [47], hence chronic use of proton-pump inhibitors could be related to small intestinal bacterial overgrowth (SIBO) [48], *Candida albicans* infections [49], and vitamin B12 deficiency in some population groups such as the elderly [50].

The above together with the new findings about involved pathways pH-independent in GI injury caused by NSAIDs, lead us to think about different strategies than PPI to prevent GI damage cause by NSAIDs.

In a previous review [51] we summarized how melatonin (MLT), as an antioxidant molecule, inhibits inflammation processes associated to different illnesses. The link between enterocyte redox state and NSAIDs GI pathology encouraged us to study if MLT administration would affect in some way. To evaluate the role of MLT in GI injury prevention induced by NSAIDs, SD was selected as GI damage model because this NSAID has a pKa of 4.15 [52] and as explained before, there is a highly significant inverse correlation between pKa and concentration required for maximum stimulation of mitochondrial oxidative stress. The higher the pKa value, the lower concentration required for maximum uncoupling of mitochondrial oxidative phosphorylation [31]. When comparing with other NSAIDs, such as acetylsalicylic acid (ASA) whose pKa is 3.5, an almost four times higher concentration of ASA is necessary to produce the same effect as SD. Furthermore, other authors studied the role of MLT to recover intestinal permeability after a SD treatment [53], so SD is a proper model of GI injury.

The main objective of this work is to study and to evaluate the effect of MLT as preventive agent of GI injury caused by SD in two different models, ex vivo and in vivo.

## 2. Materials and Methods

### 2.1. Chemical and Reagents

MLT, SD, phosphoric acid (H_3_PO_4_), disodium hydrogen phosphate (Na_2_HPO_4_), formaldehyde, and paraffin wax were purchased from Sigma-Aldrich (Madrid, Spain). Potassium dihydrogen phosphate (KH_2_PO4), potassium hydroxide (KOH), methanol (MeOH), and phosphoric acid (H_3_PO_4_) were purchased from Panreac Quimica (Barcelona, Spain). Hanks’ Balanced Salt solution (HBSS), hematoxylin and eosin were purchased from Merck S.L. (Barcelona, Spain). Double-distilled water was obtained from a Milli-Q^®^ Gradient A10 system apparatus (Millipore Iberica S.A.U., Madrid, Spain). TRIZol reagent and RevertAid First Strand cDNA synthesis kit were purchased from Thermo Fisher Scientific (Barcelona, Spain).

### 2.2. Ex Vivo NSAID/ML Administration in Pig Intestine

As the previous stage of in vivo studies, we set and validated an ex vivo model [54], which allows the study of the local effect of SD and MLT in the small intestinal mucosa; at the same time, the model allows the evaluation of the intestinal apparent permeability coefficient (Papp) for SD. Papp of SD alone and in combination with MLT were compared to evaluate the influence of MLT in the intestinal absorption of SD.

Ex vivo experiments were performed as described a previous paper [54] in the duodenum, the most proximal portion of the small intestine, of young female pigs (*Sus scrofa*). Animals were sacrificed for other purposes in the Animal Facility at Bellvitge Campus (University of Barcelona, Barcelona, Spain) and intestinal samples were obtained according to the 3R (reduction, refinement and replacement) principle.

Duodenum was excised, after that cleaned and preserved in HBBS at 5 ± 3 °C for 12 h. Then 6 cm × 6 cm pieces were cut, mounted on Franz cells (Vidrafoc, Barcelona, Spain). To avoid damages in the biological intestinal membrane, 0.02 M phosphate-buffered saline (PBS) pH 7.4 was prepared as receiving media. Composition was 0.6 g of KH_2_PO_4_ and 3.17 g of Na_2_HPO_4_ per liter of double-distilled water. pH was adjusted to 7.4 with H_3_PO_4_ or NaOH. Homogeneity and simulation of intestinal conditions during experiments were ensured by a small Teflon^®^ coated magnetic stir bar at 700 revolutions per minute of rotor (rpm) corresponding to a relative centrifugal force (rcf) or G-force of 2. The diffusion cells were previously incubated in a water bath to equalize the temperature in all cells (37 ± 1 °C).

Of SD 1 mg/mL in PBS pH 7.4, alone or in combination with 0.5 mg/mL of MLT in PBS pH 7.4, were applied into the donor chamber and sealed by parafilm immediately to prevent water evaporation. After six hours of simulated permeation duodenum samples were prepared, according Section 2.4 and Section 2.5, to perform histological analysis and COX-2/iNOS determination. Different experimental group are summarized in Table 1.

#### 2.2.1. HPLC-UV Procedure and Instrumentation

The HPLC equipment consisted of a Waters^®^ Alliance 2695 Separation Module (Waters Co., Milford, MA, USA) with a 2996 Photodiode Array Detector (DAD) at a wavelength range of 190–800 nm and sensitivity settings from 0.0001 to 2.0000 absorbance units. HPLC parameters are listed below.

SD analysis was conducted with a reverse-phase column ultrabase Nova-Pak C18, 60 Å, (150 mm × 3.9 mm; diameter of 4 µm (Waters, Barcelona, Spain) with a UV detector set up at 276 nm. The mobile phase, previously filtered by a 0.45 μm nylon membrane filter (Technokroma, Barcelona, Spain) and degassed by sonication, consisted of a 68:32 ratio of MeOH and H_3_PO_4_ (pH of 3.2) under isocratic elution at a flow rate of 0.8 mL/min. The injection volume was 50 μL.

#### 2.2.2. Validation and Verification of Analytical Methods

Previously validated HPLC-UV method was selected for the analysis of SD. Considering that the samples were obtained from biological sources, the specificity was studied. Specificity, expressed by the ICH guidelines as the ability to assess an analyte in the presence of components, which may be expected to be present, was evaluated by the absence of interference of the phosphate-buffered saline (PBS; pH 7.4) and other components from biological membranes used as a blank at the retention times shown by the different standard solutions.

#### 2.2.3. Sample Analysis

The cumulative amount of SD, alone or in combination with MLT, through the small intestine membrane from the acceptor compartment was monitored by a validated HPLC-UV methodology. Results are reported as mean ± SD of six experiments (*n* = 6).

#### 2.2.4. Data Treatment and Statistical Analysis

The permeability model has the same structure as the two-compartment classic model, composed of donor (apical) and receptor (basal) chambers, both separated by the permeation membrane. So, apical-to-basal Papp was calculated based on classic parameters according to Equation (1):

Papp = (dQ/dt)/(C_0_ × A), (1)

where (dQ/dt) is the transport rate or flux (J) (µg/min) across the biological membrane, C0 (µg/mL) is the initial concentration of the drug in the donor chamber, and A is the surface area (cm^2^) of the permeation membrane.

The cumulative amount (Q; µg) permeated through porcine duodenum was obtained by multiplying the acquired concentration (µg/mL) of SD at the receptor chamber and the volume (mL) of the receptor chamber. J (µg/min) was calculated as the slope at the steady state obtained by linear regression analysis (GraphPad Prism^®^ software, v. 5.01, GraphPad Software Inc., San Diego, CA, USA) of Q as a function of time (min). Then, Papp (cm/min) was calculated according to Equation (1) by dividing the J (µg/min), the permeation area (A; 2.54 cm^2^), and the initial drug concentration (C_0_; µg/mL = µg/cm^3^) in the donor chamber. Finally, the units were expressed in centimeters per second. 

Obtained experimental data were analyzed by unpaired Student’s *t*-test to compare Papp values for both SD Papp alone and SD Papp in presence of MLT. A *p*-value < 0.05 was established as an indicator of statistically significant differences.

### 2.3. In Vivo Studies: Oral Administration of NSAID and MLT in Mice

In vivo studies were carried out in both, female and male, *Mus musculus* mice weighing 25 ± 2 g. The mice were maintained in a controlled environment at (20 ± 1) °C for 7 days with a 12-h light/dark cycle and 50% ± 5% relative humidity throughout the experimental period. All mice were allowed free access to water and chow diet.

The nasogastric administration of SD therapeutic dose was carried out once per day for 7 days alone or in combination with MLT. No drug was administered to the reference or control group. Table 2 summarizes different experimental groups:

At the end of the experiment, eighth day, mice were euthanized. The studies were conducted under a protocol approved by the Animal Experimentation Ethics Committee of the University of Barcelona (Spain) and the Committee of Animal Experimentation of the regional autonomous government of Catalonia (Spain) 491/18 approved on September 2019.

Intestine and stomach samples were prepared, according Section 2.4 and Section 2.5, to perform histological analysis, COX-2 and iNOS determination.

### 2.4. Histological Analysis

For histological observation of stomach and intestinal architecture, hematoxylin and eosin staining were performed. For pork intestines, following the intestinal permeation study in Franz diffusion cells the samples were fixed in 4% buffered formaldehyde at room temperature. Mice stomachs and intestine were excised immediately after sacrifice and fixed in 4% buffered formaldehyde at room temperature. After fixation, all samples were paraffin embedded onto cassettes, sectioned into 5 µm slices, mounted on microscope slides and stained with hematoxylin and eosin, and finally viewed under a microscope Olympus BX41 and camera Olympus XC50 (Olympus, Barcelona, Spain).

### 2.5. COX-2 and iNOS Determination

COX-2 and iNOs determination was carried out by quantitative reverse transcription PCR (RT-qPCR), with this purpose RNA isolation was firstly carried out.

#### 2.5.1. RNA Isolation

Total RNA from the intestine pig, intestine and stomach mice tissues was isolated using TRIZol^®^ method (Thermo Fisher Scientific, Barcelona, Spain). Small tissue fragments were homogenized using 1 mL of cold TRIZol reagent and 3 min under the Polytron^TM^ Homogenizer PT1200E (Thermo Fisher Scientific, Barcelona, Spain). Then, instructions described by the manufacturer were followed. RNA concentration and quality were tested by NanoDropTM 2000/2000c Spectrophotometer (Thermo Fisher Scientific, Barcelona, Spain).

#### 2.5.2. RT-qPCR

One microgram of total RNA was reverse transcribed to cDNA using the RevertAid First Strand cDNA synthesis kit. Subsequently, qPCR was performed using the Step One Plus Real Time PCR (Applied Biosystem) and primers for COX-2 (*Sus scrofa*: Forward 5′-GGAGAGACAGCATAAACTGC-3′ and Reverse 5′-GTGTGTTAAACTCAGCAGCA-3′; *Mus musculus*: Forward 5′-CCACTTCAAGGGAGTCTGGA-3′ and Reverse 5’-AGTCATCTGCTACGGGAGGA-3′) and iNOS (*Sus scrofa*: Forward 5′-CAACAATGGCAACATCAGG-3′ and Reverse 5′-CATCAGGCATCTGGTAGC-3′; *Mus musculus*: Forward 5′-GTTCTCAGCCCAACAATACAAGA-3′ and Reverse 5′-GTGGACGGGTCGATGTCAC-3′). β-actin was used as housekeeping and PCR cycling conditions included: 5 min at 94 °C for denaturalization, 30 cycles of amplification at 72 °C for 2 min, 1 min at 94 °C, 1 min at 60 °C, and a last cycle at 72 °C for 10 min for final extension. Then, Ct values of each sample were recorded, and data were analyzed by normalization to the internal control values using the formula 2-AACt. Table 3 summarizes primers sequences.

#### 2.5.3. Data Treatment and Statistical Analysis

COX-2 and iNOS results were evaluated statistically by ANOVA followed of Dunnett’s Multiple Comparison Test using GraphPad^®^ Prism 5.01 software; all the samples were compared with control one. *p* value from 0.01 to 0.05, 0.01 to 0.001, and *p* < 0.001 were considered statistically significant, very significant and extremely significant, respectively. Asterisks indicate statistical significance. (* *p* < 0.05 > 0.01; ** *p* < 0.01 > 0.001; and *** *p* < 0.001).

## 3. Results

### 3.1. Obtained Kinetics Parameters, Papp Calculation, and Statistical Analysis

**Figure 3:**
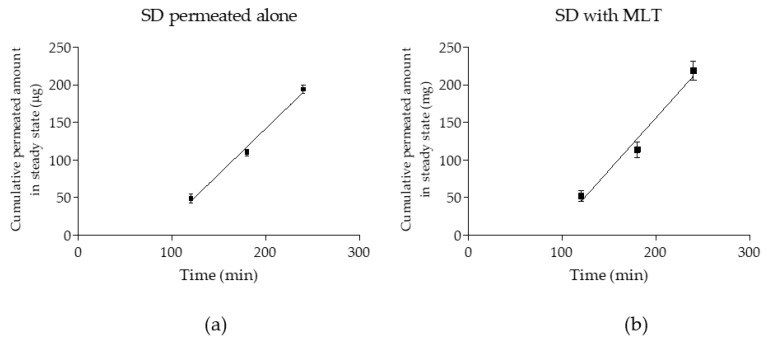
Cumulative permeated amounts (µg) as a function of time (min) in steady state of SD alone (**a**) and in the presence of melatonin (MLT) and (**b**) results are reported as mean ± SD (*n* = 6).

Figure 3 shows SD cumulative permeated amounts in micrograms as a function of time (min) in steady state, for both, SD alone and SD permeated in presence of MLT. The kinetics parameters and Papp values of SD alone and in presence of MLT and statistical correlation between both are summarized in Table 4, which shows that no statistically significant differences (*p* > 0.05) were observed correlating Papp values.

### 3.2. Histological Results: Pig Intestine and Mice Stomach and Intestine

**Figure 4:**
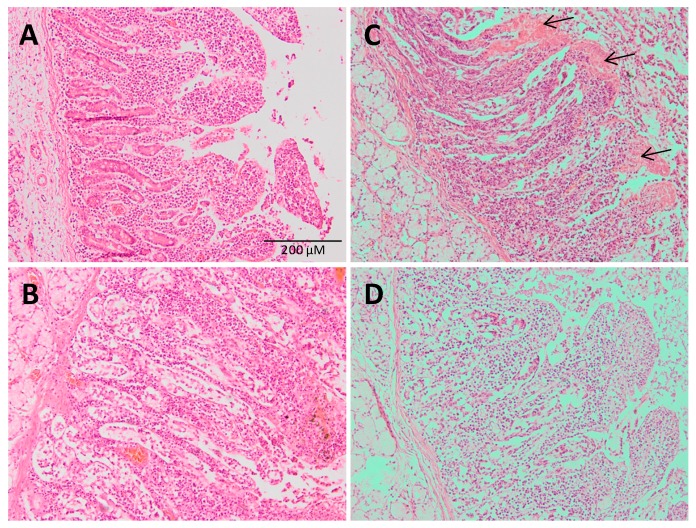
Histological analysis of intestine structure. Photomicrographs of hematoxylin- and eosin-stained sections of pig small intestine without treatment (**A**) or treated with MLT; (**B**) with SD; (**C**) or with SD + MLT; and (**D**) the effect on the histological architecture of the products was evaluated on the freshly excised pork intestine. ×100 magnification, scale bar = 200 µm. Arrow indicates alteration of villi.

To investigate the possible preventive effect of MLT when administered with SD the stomach and intestine architecture were studied. The pig intestine of the permeation ex vivo studies was stained and studied under microscope. As shown in Figure 4, a section of the small intestine without treatment (Figure 4A) showed intact layers consisting of the mucosa and the submucosa. Intestine treated with MLT (Figure 4B) showed a similar pattern of the control conditions with relatively intact villi with normal adjacent structures.

However, when the intestine was treated with SD (Figure 4C), damage to the tips of the intestinal villi could be observed. Abnormal epithelial cells are indicated with arrows. On the contrary, when the intestine was treated with SD together with MLT no relevant histopathologic changes were noticed suggesting an improvement of the intestinal injury due to SD thus preventing the damage.

**Figure 5:**
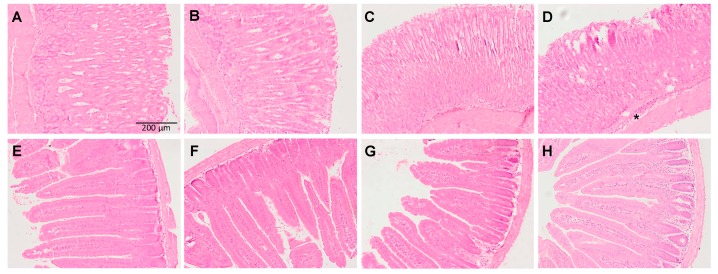
Representative micrographs of hematoxylin and eosin staining of tissue sections corresponding to the different treatments and structures: Stomach samples from mice without treatment (**A**) MLT; (**B**) SD + MLT; (**C**) or SD; (**D**) small intestine from mice without treatment; (**E**) MLT; (**F**) SD + MLT; (**G**) or SD; and (**H**) the effect on the histological architecture of the products was evaluated on the stomach and intestine from *p*.o. administered mice. ×100 magnification, scale bar = 200 µm. * indicates leucocytes infiltration.

Regarding the in vivo studies, as shown in Figure 5, section of the mice stomach without treatment (Figure 5A) showed intact layers consisting of the mucosa and the submucosa. Samples treated with MLT (Figure 5B) showed a similar pattern of the control conditions. The results show that SD altered stomach mucosa and induced tissue damage as it can be shown by the leucocyte infiltrate (Figure 5D), which was prevented when MLT was administered (Figure 5C). For the mice intestinal architecture little alterations were observed with the anti-inflammatory drug alone (Figure 5H) or in combination with MLT (Figure 5G).

### 3.3. COX-2 and iNOs Levels

**Figure 6:**
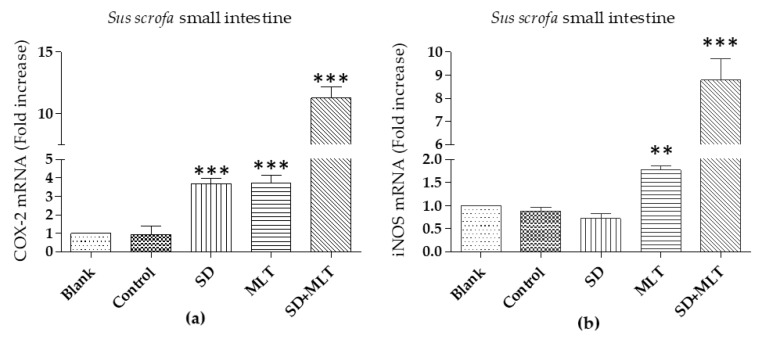
Comparative levels of COX-2 (**a**) and iNOS; and (**b**) mRNA in duodenum of *Sus scrofa.* Data are expressed as mean ± SD. ** *p* < 0.01 > 0.001; and *** *p* < 0.001. SD (Sodium diclofenac); MLT (Melatonin).

COX-2 and iNOs obtained levels in duodenum samples of *Sus scrofa* are reported in Figure 6, which shows that comparison between iNOS levels in samples corresponding to MLT and control resulted in statistically very significant differences (** *p* < 0.01 > 0.001) and extremely significant (*** *p* < 0.001) in the case of SD with MLT samples. On behalf of COX-2 extremely significant differences (*** *p* < 0.001) were observed for SD, MLT, and SD with MLT.

**Figure 7:**
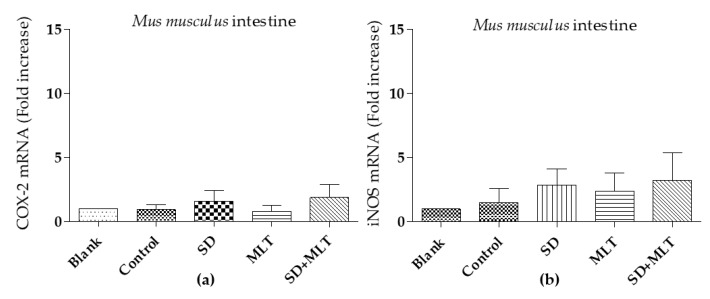
Comparative levels of COX-2 (**a**) and iNOS; and (**b**) mRNA in duodenum of *Mus musculus.* Data are expressed as mean ± SD. MLT (Melatonin); SD (Sodium diclofenac).

**Figure 8:**
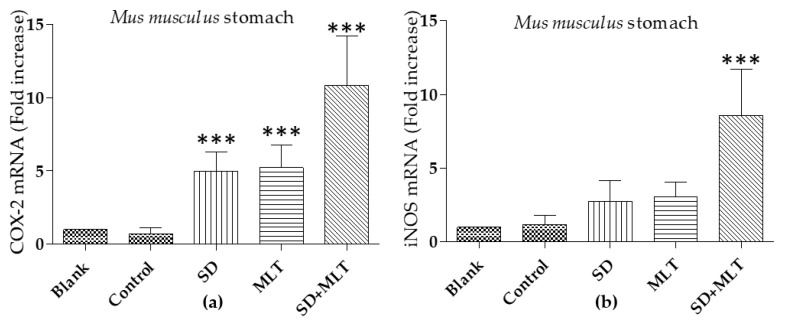
Comparative levels of COX-2 (**a**) and iNOS; and (**b**) mRNA in the stomach of *Mus musculus.* Data are expressed as mean ± SD. *** *p* < 0.001. SD (Sodium diclofenac); MLT (Melatonin).

COX-2 and iNOs obtained levels for *Mus musculus* duodenum (intestine) and stomach are summarized in Figure 7 and Figure 8 respectively. Concerning mice intestine no differences statistically significant (*p* > 0.05) were observed for different samples, for both COX-2 and iNOS levels.

Observed levels for COX-2 in *Mus musculus* stomach samples for SD, MLT, and SD with MLT were extremely significant (*** *p* < 0.001) different from control. With regard to iNOS only SD with MLT samples lead to extremely significant (*** *p* < 0.001) differences when compared to control.

## 4. Discussion

Differences between gastric and intestinal damages caused by SD for an in vivo study are related to the pH of the tissue and the pKa the NSAID. As previously discussed, there is a highly significant inverse correlation between pKa and concentration required for maximum stimulation of mitochondrial oxidative stress, the lower pH of the stomach together with increased acid secretion and decreased gastric fluid volume cause by SD may lead in higher damages in mice stomach than in small intestine.

**Figure 9:**
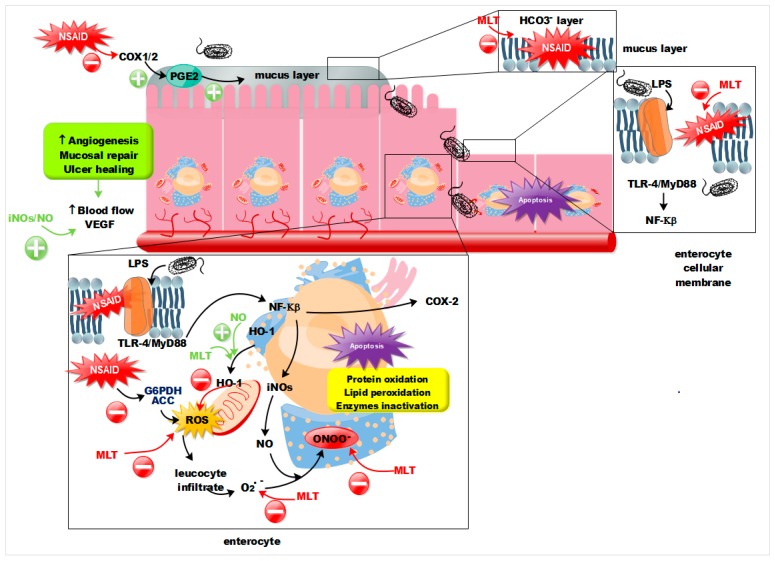
Summarized MLT multipathways action mechanism to prevent GI injuries caused by NSAIDs.

As summarized in Figure 9, the observed prevention of GI damage caused by MLT may be involved in multiple pathways.

The amphiphilic nature of MLT, which has lipophilic [55] and hydrophilic groups [56], allows it to interact with PC based membranes [57] or lipidic membranes [58], in fact MLT crosses all the biological membranes including the blood brain barrier [59] and as a result of such an interaction MLT shows a role in preserving the integrity, permeability, and functionality of cell membranes [60,61], therefore at the GI level MLT may interact with enterocyte cellular membrane decreasing the NSAIDs damage. Additionally, MLT showed a stimulant effect of mucosal bicarbonate secretion [62], so MLT may also protect the HCO_3_^−^ mucus layer.

The raised levels of iNOS in samples treated with MLT together with SD are indicative of higher NO expression; NO may be intermediate of mucosal repair by increasing angiogenesis and blood flow, since MLT improves enterocyte redox state at different levels working as scavenger of ROS (O2^−^), avoiding the interaction between NO and ROS, and ONOO^−^ production ultimately. Furthermore, increased levels of COX-2 in mice samples treated with MLT together with SD are indicative of increased levels of PGI_2_, which inhibits the in vivo gastric acid secretion and enhances mucosal blood flow [10] that would add to the effect of NO on the inhibition of gastric acid secretion [35].

An alternative mechanism to the scavenger action of MLT protective agent is related with the heme oxygenase 1 (HO-1), this enzyme encoded by the *Hmox1* gene, is 32-kDa stress protein induced in human cells by a variety of stress treatments. [63] The induction of this enzyme is part of a protective response against oxidative damage via down-regulation of reactive oxygen species generation [64]. The translocation from endoplasmic reticulum to mitochondria of HO-1 was shown as a novel cytoprotective mechanism against NSAIDs drug-induced oxidative stress, apoptosis, and gastric mucosal injury [65] and MLT was proved to be an enhancer of HO-1 activity [66] so one of the cellular pathways involved in the protective effect of MLT against GI mucosal damage may be related with the enhancement of this protective enzyme.

Increased levels of COX-2 in samples where MLT prevented SD damages, may be related with the wound healing effect of COX-2 in enterocytes [17] and with an increased mucosal blood flow [10].

Results show the role of MLT in tissue damage prevention at intestinal level in the ex vivo model and at gastric level in the in vivo experiment. Despite of higher levels of COX-2 and iNOS in the samples treated with MLT together with SD, they showed a prevention of GI damage, as discussed, this prevention is mainly due to the antioxidant effect of MLT that improves and contributes to a favorable redox status of enterocyte; although the increasing mechanism for both enzymes is unclear, because other authors relate MLT to the inhibition of COX-2 and iNOS in different cells (murine macrophage cell line, RAW 264.7) [67]. With respect to the preventive effect, all of our results of the individuals within a group were homogenous and male and female results were considered together, however some authors found that sexual hormones could influence the healing process of preexisting ulcers [68]. Other study showed that ovarian sex hormones neither worsen nor protect against aspirin-induced gastric lesions in female rats [69].

Permeation results show that MLT would slightly increase SD Papp but according to statistical correlation, the difference is not statistically significant (*p* > 0.05), therefore a joint administration of both drugs may not affect SD absorption.

## 5. Conclusions

Our investigation contributes the study of the effect of MLT as a preventive agent of GI damage caused by SD in an ex vivo model and the study of the effect of MLT as a preventive agent of GI damage when administered jointly to SD over a period of 7 days in a mice in vivo model.

MLT showed a preventive effect of GI lesions induced by SD. Numerous pathways would outline as responsible for this effect, the direct inhibition of ROS and as consequence the inhibition of all the derived molecules that cause GI damage, the enhancement of mitochondrial protection factor HO-1 and a protective role on the phospholipids as PC of the GI membrane and mucus layer.

Future research could focus on a deeply study of MLT as an alternative to classical therapies based on PPI in the prevention of GI damage caused by NSAIDs.

## Figures and Tables

**Table 1:** Ex vivo experimental groups.

Group Number	Permeated Drug	Permeated Solution	Dose
1 (*n* = 6)	No drug (Reference group)	-	-
2 (*n* = 6)	SD	1 mg/mL in PBS pH 7.4	1 mL
3 (*n* = 6)	MLT	0.5 mg/mL in PBS pH 7.4	1 mL
4 (*n* = 6)	SD (a) + MLT (b)	2 mg/mL (a) 1 mg/mL (b)	0.5 mL + 0.5 mL

SD (Sodium Diclofenac); MLT (Melatonin).

**Table 2:** In vivo experimental groups.

Group number	Group	Administered Drugs	Dose
1 (*n* = 6)	REF	No drug (Reference group)	-
2 (*n* = 6)	SD	Sodium Diclofenac	2.5 mg/kg
3 (*n* = 6)	MLT	Melatonin	10 mg/kg
4 (*n* = 6)	SD + MLT	Sodium Diclofenac and Melatonin	2.5 mg/kg and 10 mg/kg

REF (Reference group); SD (Sodium Diclofenac); MLT (Melatonin).

**Table 3:** Primer sequences used for real time PCR in *Sus scrofa* and *Mus musculus*.

Primer	Animal Model	Sequence (5′ to 3′)	Acc. Number
COX-2	*Sus scrofa*	FW: GGAGAGACAGCATAAACTGC	AF207824
		RV: GTGTGTTAAACTCAGCAGCA	
	*Mus musculus*	FW: CCACTTCAAGGGAGTCTGGA	NM_011198.4
		RV: AGTCATCTGCTACGGGAGGA	
iNOS	*Sus scrofa*	FW: CAACAATGGCAACATCAGG	U59390
		RV: CATCAGGCATCTGGTAGC	
	*Mus musculus*	FW: GTTCTCAGCCCAACAATACAAGA	NM_010927
		RV: GTGGACGGGTCGATGTCAC	
β-actin	*Sus scrofa*	FW: GACATCCGCAAGGACCTCTA	DQ845171
		RV: ACACGGAGTACTTGCGCTCT	
	*Mus musculus*	FW: GGCCGGGACCTGACAGACTACCTC	NM_007393
		RV: GTCACGCACGATTTCCCTCTCAGC	

**Table 4:** Permeation parameters for SD alone and in presence of MLT in vertical Franz cells (*n* = 6) and statistical correlation between different Papp obtained values. Papp values are expressed as mean ± SD.

Permeation Parameters	SD Alone	SD+MLT	Unpaired *t*-Test (*p*)
**Flux (µg/min)**	1.21 ± 0.11	1.39 ± 0.20	-
**Flux/sup (µg/(cm/min))**	0.48 ± 0.04	0.55 ± 0.10	-
**Co (µg/mL)**	1000	1000	-
**P_app_ (×10^−6^) (cm/s)**	7.92 ± 0.73	9.12 ± 0.13	0.078 (>0.05)

Abbreviation: P_app_—apparent permeability coefficient; SD—Sodium Diclofenac; MLT: Melatonin.
